# Replication of the genetic effects of IFN regulatory factor 5 (*IRF5*) on systemic lupus erythematosus in a Korean population

**DOI:** 10.1186/ar2152

**Published:** 2007-03-27

**Authors:** Hyoung Doo Shin, Yoon-Kyoung Sung, Chan-Bum Choi, Soo Ok Lee, Hye Won Lee, Sang-Cheol Bae

**Affiliations:** 1 Department of Genetic Epidemiology, SNP Genetics Inc., Rm 1407, 14th floor, Complex B, WooLim Lion's Valley, 371-28, Gasan-Dong, Geumcheon-Gu, Seoul 153-801, Korea; 2 Department of Internal Medicine, Division of Rheumatology, Hospital for Rheumatic Diseases, Hanyang University, 17 Hangdang Dong, Sungdong-Gu, Seoul 133-792, Korea

## Abstract

Recently, two studies provided convincing evidence that IFN regulatory factor 5 (*IRF5*) gene polymorphisms are significantly associated with systemic lupus erythematosus (SLE) in several white populations. To replicate the association with SLE in an Asian population, we examined the genetic effects in our SLE cohort from a Korean population. A total of 1,565 subjects, composed of 593 cases and 972 controls, were genotyped using the TaqMan^® ^(Applied Biosystems, Foster City, CA, USA) method. The genetic effects of polymorphisms on the risk of SLE were evaluated using χ^2 ^tests and a Mantel–Haenszel meta-analysis. Statistical analysis revealed results in the Korean population were similar to the previous reports from white populations. The rs2004640 T allele had a higher frequency in SLE cases (0.385) than controls (0.321; odds ratio (OR) = 1.32, *P *= 0.0003). In combined analysis, including all seven independent cohorts from the three studies so far, robust and consistent associations of the rs2004640 T allele with SLE were observed. The estimate of risk was OR = 1.44 (range, 1.34–1.55), with an overall *P *= 1.85 × 10^-23 ^for the rs2004640 T allele. The haplotype (rs2004640T–rs2280714T) involved in both the alternative splice donor site and the elevated expression of IRF5 also had a highly significant association with SLE (pooled, *P *= 2.11 × 10^-16^). Our results indicate that the genetic effect on the risk of SLE mediated by IRF5 variants can be generally accepted in both white and Asian populations.

## Introduction

Recently, two studies provided convincing evidence that IFN regulatory factor 5 (*IRF5 *[MIM 607218]) gene polymorphisms are significantly associated with systemic lupus erythematosus (SLE [MIM 152700]). The studies included – seven independent SLE cohorts from white populations (Sweden-1, Finland, Iceland, USA, Spain, Sweden-2 and Argentina) and involved both family-based and case-control cohorts [1,2]. In both studies, the dbSNP rs2004640 (T > G) of *IRF5 *showed strong associations with the risk of SLE, for example higher frequencies in SLE cases than controls (combined analysis, 61% in SLE cases versus 51% in controls; *P *= 4.2 × 10^-21^). Graham and colleagues, through further experiments in the later study, also identified a common (frequency, 50.0% in white populations) *IRF5 *haplotype that has both a splice donor site, which allows expression of multiple IRF5 isoforms containing exon 1B, and a separate genetic effect associated with elevated levels of expression of IRF5 [1]. To replicate the association with SLE in an Asian population, we examined the genetic effects in our SLE cohort from a Korean population.

## Materials and methods

A total of 593 Korean SLE patients (mean age, 32.36 (6.99–70.7); male = 35 and female = 558) who fulfilled the 1997 American College of Rheumatology (ACR) criteria for SLE [3] were consecutively enrolled between September 1998 and February 2005 at the Hospital for Rheumatic Diseases, Hanyang University, Seoul, Korea. The following clinical and laboratory data were obtained: sex, age, ages at onset of first symptom and clinical diagnosis, ACR diagnosis, and Systemic Lupus International Collaborating Clinics (SLICC)/ACR damage index [4]. As a control group, we included 971 healthy, ethnic-matched subjects (mean age, 37.2 (16.6–78.6); male = 139 and female = 832).

Four SNPs (rs729302 (A > C), rs2004640 (G > T), rs752637 (T > C) and rs2280714 (T > C)) were genotyped, using the TaqMan^® ^(Applied Biosystems, Foster City, CA, USA) method [5], in our SLE cases and controls from the Korean population. Information regarding the primers is available on our website [6].

χ^2 ^analyses were used to evaluate the significance of differences in genotype and allele frequencies in the case-control samples using Statistical Analysis System (SAS). The allele frequencies for cases and controls were used to calculate the odds ratio (OR) and the 95% confidence interval using SAS. For the case-control haplotype analysis, Haploview v3.2 (Broad Institute of Harvard and MIT Cambridge, MA, USA) was used to generate haplotype frequencies and calculate the significance of associations. The Breslow–Day statistic was used to test for homogeneity among studies. A Mantel–Haenszel meta-analysis was performed on the ORs, and these data were subsequently combined in a separate analysis with the published results of the association of rs2004640 (G > T) with SLE from previous studies [1,2]. Further conditional analyses and global haplotype tests were performed using WHAP software [7] developed by Shaun Purcell (Massachusetts General Hospital, Boston, MA, USA) and Pak Sham (Hong Kong University, Hong Kong). This analysis was used to disentangle the correlation structure in the gene, to rule out the possibility that multiple observed effects are owing to linkage disequilibrium from a single true effect.

## Results and discussion

Genotype distributions of all loci were in Hardy–Weinberg equilibrium (*P *> 0.05). The frequency of the rs2004640 T allele, which has a central role in *IRF5 *polymorphisms, was significantly lower in the Korean population (frequency, 0.345) than white populations (frequency, 0.570; Table S2 in Additional file 1).

Statistical analysis revealed the results in the Korean population were similar to the previous reports from white populations [1,2]. The rs2004640 T allele was significantly associated with an increased risk of SLE (Table 1), for example it had a higher frequency in SLE cases (0.385) than controls (0.321; OR = 1.32, *P *= 0.0003). The two nearby SNPs (rs729302 and rs752637) that were strongly linked to rs2004640 (|D'| = 0.83 and 0.98, respectively; Figure S1 in Additional file 1) and the haplotype (rs2004640T–rs2280714T) that was associated with both the alternative splice donor site and elevated levels of expression of IRF5 [1] were also significantly associated with SLE (Table 1 and Table S1 in Additional file 1).

**Table 1:** Allele/haplotype distribution of *IRF5 *polymorphisms in Korean SLE patients/controls and association analysis for SLE

Loci	Associated allele	Cases	Controls	OR (95% CI)	χ^2^	*P*^a^
		(*n *= 593)	(*n *= 972)			
rs729302 (A > C)	A	0.729	0.680	1.27 (1.08–1.49)	8.44	0.0037
rs2004640 (G > T)	T	0.385	0.321	1.32 (1.14–1.54)	13.22	0.0003
rs752637 (T > C)	C	0.455	0.398	1.27 (1.09–1.47)	9.73	0.0018
rs2280714 (T > C)	T	0.395	0.402	0.97 (0.84–1.13)	0.15	0.6971
	
Haplotype^b^	AGTC	0.355	0.368	0.95 (0.82–1.11)	0.49	0.4840
	ATCT	0.352	0.295	1.28 (0.10–1.50)	10.85	0.001
	CGTT	0.158	0.205	0.74 (0.61–0.89)	10.21	0.0014
	CGCT	0.066	0.071	0.96 (0.72–1.28)	0.34	0.5600
	CGTC	0.034	0.024	0.97 (0.63–1.50)	0.78	0.3784
	CTCT	0.016	0.017	1.23 (0.72–2.09)	0.07	0.7919
Haplotype^c^	T–T	0.380	0.311	1.36 (1.16–1.58)	15.2	9.64 × 10^-5^
	G–T	0.225	0.283	0.74 (0.62–0.87)	12.5	0.0004
	G–C	0.387	0.398	0.95 (0.82–1.11)	0.4	0.5330
	T–T	0.008	0.008	0.96 (0.42–2.21)	0.0	0.9323

Case-control analysis. χ^2 ^analyses were used to evaluate the significance of differences in genotype and allele frequencies in the case-control samples. The allele frequencies for cases and controls were used to calculate the OR and the 95% CI. For the case-control haplotype analysis, Haploview v3.2 (Broad Institute of Harvard and MIT Cambridge, MA, USA) was used to generate haplotype frequencies and calculate the significance of associations. CI, confidence interval; IRF5, IFN regulatory factor 5; OR, odds ratio; SLE, systemic lupus erythematosus.

^a^*P *value, uncorrected for multiple tests.

^b^Haplotype consisting of markers rs729302 (A > C), rs2004640 (G > T), rs752637 (T > C) and rs2280714 (T > C).

^c^Haplotype consisting of markers rs2004640 (G > T) and rs2280714 (T > C).

The observation that the presence of risk haplotypes within datasets can create spurious protective effects for other haplotypes lead us to perform the same global test conditioning for the predisposing haplotype (T–T). Conditioning for the predisposing haplotype (T–T) revealed that no significant haplotypic association remained in the dataset (χ^2 ^(2 degrees of freedom) = 5.456, *P *= 0.07). These results might suggest that the risk haplotype (T–T) could explain the total association, for example the protective effect of the haplotype (G–T) was not real and could be just a shadow effect of the risk haplotype (T–T).

In combined analysis, including all eight independent cohorts (Finland, Iceland, USA, Spain, Sweden-1, Sweden-2, Argentina and Korea) from the three studies so far, robust and consistent associations of the rs2004640 T allele with SLE were observed (Table 2). The Breslow–Day test for heterogeneity was not significant for allele distributions (*P *= 0.7115, data not shown), suggesting the homogeneity of studies. The estimate of risk was OR = 1.44 (1.34–1.55), with an overall *P *= 1.85 × 10^-23 ^for the rs2004640 T allele (Table 2). The haplotype (rs2004640T–rs2280714T) involved in both the alternative splice donor site and the elevated levels of expression of IRF5 [1] also had a highly significant association with SLE (pooled, *P *= 2.11 × 10^-16^; Table S1 in Additional file 1). The strengths of the associations of both the rs2004640 T allele and the haplotype were high enough to surpass the correction for multiple testing, even with all of the variants in the human genome.

**Table 2:** Case-control association analysis of the *IRF5 *rs2004640 (G > T) T allele with SLE

		n	No. of T alleles	Frequency of T alleles	No. of G alleles	Frequency of G alleles	OR (95% CI)	*P*^a^	Pooled OR^b^	Pooled *P*^b^
Korea	Cases	589	454	0.385	724	0.615	1.32 (1.14–1.54)	0.0003		
	Controls	950	610	0.321	1290	0.679				

Argentina^c^	Cases	284	309	0.54	259	0.46	1.52 (1.20–1.93)	0.00035		
	Controls	279	245	0.44	313	0.56				
										
Spain^c^	Cases	444	559	0.63	329	0.37	1.42 (1.18–1.71)	0.00016		
	Controls	541	589	0.54	493	0.46				
									1.45 (1.32–1.58)	4.4 × 10^-16^
										
Sweden-1^c^	Cases	208	260	0.63	156	0.38	1.31 (1.01–1.71)	0.04268		
	Controls	254	284	0.56	224	0.44				
										
USA^c^	Cases	725	879	0.61	571	0.39	1.47 (1.29–1.67)	3.6 × 10^-9^		
	Controls	1,434	1,467	0.51	1401	0.49				

Sweden-2^d^	Cases	480	595	0.62	365	0.38	1.51 (1.21–1.87)	0.0002		
	Controls	256	266	0.52	246	0.48				
									1.59 (1.31–1.94)	7.1 × 10^-7^
										
Finland^d^	Cases	109	137	0.63	81	0.37	1.84 (1.27–2.66)	0.00133		
	Controls	121	116	0.48	126	0.52				

Combined analysis	Cases	2,839	3,193	0.56	2,485	0.44			1.44 (1.34–1.55)	1.85 × 10^-23^
	Controls	3,835	3,577	0.47	4,093	0.53				

Meta-analysis. Mantel–Haenszel meta-analysis of the ORs; these data were subsequently combined in a separate analysis with the published results of the association of rs2004640 (G > T) with SLE from previous studies [1,2]. The Breslow–Day test for heterogeneity was not significant for allele distributions (*P *= 0.7115, data not shown), suggesting the homogeneity of studies. 'Number of alleles' refers to number of alleles of rs2004640 (G > T). CI, confidence interval; IRF5, IFN regulatory factor 5; OR, odds ratio; SLE, systemic lupus erythematosus.

^a^χ^2 ^tests were used to evaluate the significance of differences in allele frequencies in the case-control samples.

^b^Mantel–Haenszel test [12] of pooled ORs and 95% CIs.

^c^Data from Graham and co-workers [1].

^d^Data from Sigurdsson and co-workers [2].

Genetic association studies provide a potentially powerful tool for identifying genetic variations that influence susceptibility to common diseases. However, there are numerous cases of associations that cannot be replicated afterwards, which have led to skepticism about genetic epidemiology studies of complex diseases [8-10]. To discourage false-positive association hypotheses, several recommendations have been suggested: large sample sizes, small *P *values, a gene/allele with biologically/physiologically meaningful sense, an association observed in both family- and population-based studies, replications in independent studies, and a high OR and/or attributable risk [8]. Among these criteria, validation of a genetic association by replication might be the most important step to exclude false-positive associations. In statistical terms, independent replication decreases the chances of reporting an association if no association actually exists (type I error). In the case of association of the IRF5 variant with SLE, which has satisfied most of the above criteria and been replicated among numerous white populations, additional replications among separate sets of patients with different ethnic backgrounds, such as African and/or Asian populations, would strengthen the confidence in any association study and allow significant gains in narrowing the disease associated interval owing to different patterns of linkage disequilibrium. In this study of a Korean SLE cohort, we present associations of IRF5 variants with SLE similar to those suggested by previous studies.

Transcription factors of the IRF family have essential roles in the regulation of genes induced by viral infection and immunostimulation, in addition to regulation of cell growth. IRF5 was originally identified as a regulator of type I IFN gene expression [11]. IRF5 is regulated by type I IFN, indicating an important regulatory pathway for the controlled induction of multiple immunomodulatory genes. The constitutive expression of *IRF5 *is limited to lymphoid organs, dendritic cells and peripheral blood lymphocytes; however, it is absent in numerous leukaemia and lymphoma cell lines [11], which might indicate a propensity for *IRF5 *gene deletion or possibly silencing by methylation in these malignancies [11]. IRF5 is phosphorylated in cells on viral infection and translocates to the nucleus, which results in activation of a spectrum of IFN genes [11]. Thus, polymorphism within the *IRF5 *gene might affect several cellular functions of importance for the development of an autoimmune disease, such as SLE. The rs2004640 T allele creates a 5' donor splice site in an alternate exon 1 of IRF5 and might thus have a functional role by altering the splicing of exon 1 of the *IRF5 *[2].

## Conclusion

Association analysis of *IRF5 *polymorphisms revealed that the results in the Korean population were similar to those in the previous reports from white populations, for example the rs2004640 T allele of *IRF5 *revealed a susceptible effect on the risk of SLE. These results indicate that the genetic effect on the risk of SLE mediated by *IRF5 *variants could be generally accepted in both white and Asian populations.

## Abbreviations

ACR = American College of Rheumatology; dbSNP = database of SNP; IFN = interferon; IRF5 = IFN regulatory factor 5; OR = odds ratio; SAS = statistical analysis system; SLE = systemic lupus erythematosus; SLICC = Systemic Lupus International Collaborating Clinics; SNP = single nucleotide polymorphism.

## Competing interests

We have no competing interests (political, personal, religious, ideological, academic, intellectual, commercial or any other) to declare in relation to this manuscript.

## Authors' contributions

HD Shin and SC Bae have made substantial contributions to study design, acquisition of data, drafting the manuscript, and analysis and interpretation of data. YK Sung and CB Choi have been involved in drafting the manuscript or critically revising it. HW Lee carried out the molecular genetic studies, including genotyping. SO Lee performed the statistical analysis. All authors read and approved the final manuscript.

## Supplementary Material

Additional file 1 A DOC file containing Figure S1, which depicts LDs among *IRF5 *polymorphisms in a Korean population (cases and controls). Click here for file

Additional file 2 A DOC file containing Table S1, which shows *IRF5 *haplotype frequency in SLE cases and controls. Click here for file

Additional file 3 A DOC file containing Table S2, which shows frequencies of *IRF5 *polymorphisms and deviation from the Hardy–Weinberg equilibrium in a Korean population. Click here for file
