# Supplementary figures and images for: Replication of the genetic effects of IFN regulatory factor 5 (IRF5) on systemic lupus erythematosus in a Korean population
Source: Arthritis Res Ther. 2007 Mar 27;9(2):R32. doi: 10.1186/ar2152 (PMC1906810; doi:10.1186/ar2152)

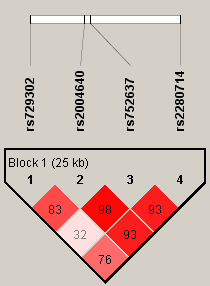

Supplement: Additional file 1 — A DOC file containing Figure S1, which depicts LDs among IRF5 polymorphisms in a Korean population (cases and controls). [file ar2152-S1.doc]
