# Supplementary material for: Replication of the genetic effects of IFN regulatory factor 5 (IRF5) on systemic lupus erythematosus in a Korean population
Source: Arthritis Res Ther. 2007 Mar 27;9(2):R32. doi: 10.1186/ar2152 (PMC1906810; doi:10.1186/ar2152)
Supplement: Additional file 2 — A DOC file containing Table S1, which shows IRF5 haplotype frequency in SLE cases and controls. [file ar2152-S2.doc]

|  | Haplotype^a^ | |  | Cases | Frequency | Controls | Frequency | OR (95% C.I.) | χ2 | *P*^b^ |
| --- | --- | --- | --- | --- | --- | --- | --- | --- | --- | --- |
|  | *rs2004640(G>T)* | *rs2280714(T>C)* |  |
|  |  |  |  | N=582^c^ |  | N=936 |  |  |  |  |
| Korea | T | T |  | 442 | 0.380 | 582 | 0.311 | 1.36(1.16-1.58) | 15.2 | 9.64X10^-5^ |
|  | G | T |  | 262 | 0.225 | 530 | 0.283 | 0.74(0.62-0.87) | 12.5 | 0.0004 |
|  | G | C |  | 450 | 0.387 | 745 | 0.398 | 0.95(0.82-1.11) | 0.4 | 0.5330 |
|  | T | C |  | 9 | 0.008 | 15 | 0.008 | 0.96(0.42-2.21) | 0.0 | 0.9323 |
|  |  |  |  | N=282 |  | N=262 |  |  |  |  |
| Argentina | T | T |  | 303 | 0.54 | 227 | 0.43 | 1.52(2.20-1.93) | 11.8 | 0.0006 |
|  | G | T |  | 54 | 0.1 | 70 | 0.13 | 0.69(0.47-1.00) | 3.8 | 0.0501 |
|  | G | C |  | 205 | 0.36 | 224 | 0.43 | 0.77(0.60-0.98) | 4.7 | 0.0309 |
|  |  |  |  | N=350 |  | N=527 |  |  |  |  |
| Spain | T | T |  | 419 | 0.6 | 547 | 0.52 | 1.38(1.14-1.68) | 10.8 | 0.001 |
|  | G | T |  | 109 | 0.16 | 167 | 0.16 | 0.98(0.79-1.27) | 0 | 0.9212 |
|  | G | C |  | 155 | 0.22 | 316 | 0.3 | 0.66(0.53-0.83) | 13.2 | 0.0003 |
|  | T | C |  | 17 | 0.02 | 25 | 0.02 | 1.02(0.55-1.91) | 0 | 0.9366 |
|  |  |  |  | N=82 |  | N=93 |  |  |  |  |
| Sweden | T | T |  | 99 | 0.6 | 109 | 0.59 | 1.08(0.70-1.65) | 0.1 | 0.7514 |
|  | G | T |  | 29 | 0.18 | 32 | 0.17 | 1.03(0.60-1.80) | 0.2 | 0.6642 |
|  | G | C |  | 36 | 0.22 | 44 | 0.24 | 0.91(0.55-1.50) | 0 | 0.9431 |
|  |  |  |  | N=649 |  | N=1405 |  |  |  |  |
| USA. | T | T |  | 780 | 0.6 | 1422 | 0.51 | 1.47(1.29-1.68) | 32 | 1.6x10^-8^ |
|  | G | T |  | 162 | 0.13 | 413 | 0.15 | 0.83(0.68-1.01) | 3.5 | 0.0599 |
|  | G | C |  | 348 | 0.27 | 961 | 0.34 | 0.71(0.61-0.82) | 22.1 | 2.6x10^-6^ |
|  |  |  |  | N=1940 |  | N=3215 |  |  |  | Pooled P^d^ |
| ALL | T | T |  | 2043 | 0.53 | 2887 | 0.449 | 1.41(1.30-1.53) | 67.5 | 2.11X10^-16^ |
|  | G | T |  | 616 | 0.16 | 1212 | 0.188 | 0.81(0.72-0.90) | 15.3 | 8.98X10^-5^ |
|  | G | C |  | 1194 | 0.31 | 2290 | 0.356 | 0.79(0.72-0.86) | 29.9 | 4.49X10^-8^ |
|  | T | C |  | 26 | 0.01 | 40 | 0.006 | 1.00(0.61-1.65) | 0.0 | 0.9925 |

Adapted from Supplementary Table 3 of Graham et al.[1]

^a^ Haplotype of *rs2004640 (G>T)*and *rs2280714 (T>C)*, phased using Haploview software. Individuals with missing genotype were excluded in haplotype analysis.

^b^ P value, uncorrected for multiple tests, 1 degree of freedom

^c^ Number of individuals

^d^ Pooled P value from Mantel-Haenszel test of pooled odds ratios [10]
