# Supplementary material for: Replication of the genetic effects of IFN regulatory factor 5 (IRF5) on systemic lupus erythematosus in a Korean population
Source: Arthritis Res Ther. 2007 Mar 27;9(2):R32. doi: 10.1186/ar2152 (PMC1906810; doi:10.1186/ar2152)
Supplement: Additional file 3 — A DOC file containing Table S2, which shows frequencies of IRF5 polymorphisms and deviation from the Hardy–Weinberg equilibrium in a Korean population. [file ar2152-S3.doc]

| #rs | Region | Genotype | | | Allele | Frequency |  | HWE^a^ | | |
| --- | --- | --- | --- | --- | --- | --- | --- | --- | --- | --- |
|  |  |  |  |  |  | Korean (n=1,565) |  | Case | Control | All |
| *rs729302(A>C)* | Promoter | A | AC | C | A | 0.699 |  | 0.271 | 0.319 | 0.743 |
|  |  | 763 | 650 | 144 |  |  |  |  |  |  |
| *rs2004640(G>T)* | Promoter | G | GT | T | T | 0.345 |  | 0.385 | 0.321 | 0.075 |
|  |  | 676 | 664 | 199 |  |  |  |  |  |  |
| *rs752637(T>C)* | Promoter | T | CT | C | C | 0.420 |  | 0.456 | 0.399 | 0.097 |
|  |  | 537 | 724 | 290 |  |  |  |  |  |  |
| *rs2280714(T>C)* | 3'down | T | CT | C | T | 0.600 |  | 0.395 | 0.402 | 0.833 |
|  |  | 566 | 747 | 252 |  |  |  |  |  |  |

^a^ P values for deviation of genotype deviation from Hardy-Weinberg equilibrium in Korean population
